# Decrease of DHEA-S concentration succeeding a micro-dose thumb exertion: mood-state determinants reflect stress-biomarker responses

**DOI:** 10.1186/s40064-016-3131-3

**Published:** 2016-08-30

**Authors:** Adam Michael Szlezak, Lotti Tajouri, Siri Lauluten Szlezak, James Keane, Clare Minahan

**Affiliations:** 1 Griffith Sports Physiology, Griffith University, Gold Coast, QLD 4215 Australia; 2 Faculty of Health Sciences and Medicine, Bond University, Robina, QLD 4226 Australia

**Keywords:** Affect, Biomarkers, Dehydroepiandrosterone, Exercise, Interleukin-6, Weight lifting

## Abstract

**Background:**

The present study examined the effect of a micro-dose of resistance-exercise on serum DHEA-S, IL-6 and mood-state determinants. Potential relationships between mood and the biomarkers were also studied with the aim of directing research on non-invasive exercise-monitoring methods.

**Methods:**

30 male participants (20 weightlifting-trained; 10 untrained) were separated into 3 groups of 10: weightlifting experimental (WL_EXP_); untrained experimental (UT_EXP_); weightlifting placebo (WL_PLA_). WL_EXP_ and UT_EXP_ performed four 60-s isometric thumb exertions separated by 60-s rest intervals in a single-blinded placebo-controlled study. Participants were assessed over a 60-min post-intervention recovery period for changes in serum DHEA-S and IL-6, and mood-state determinants (vigour, tension, fatigue).

**Results:**

DHEA-S changed in UT_EXP_ only; a decrease from 20- to 60-min post-exercise (Δ36.9 %, p < 0.01). DHEA-S remained below baseline at the final time-point (Δ35.3 %, p = 0.012). Tension decreased immediately post-exercise in WL_EXP_ (Δ86.7 %, p = 0.022), whereas UT_EXP_ showed a delayed decrease which continued up to 60-min post-intervention (Δ100 %, p < 0.01). Relative to fatigue scores recorded immediately post-exercise, WL_EXP_ decreased within the first 10-min post-intervention (Δ22.2 %, p < 0.01) whereas UT_EXP_ showed a delayed decrease evident at 20-min post-intervention (Δ25 %, p < 0.01). Serum IL-6 and vigour scores remained unchanged across groups (p > 0.05) and WL_PLA_ did not change for any measured variable (p > 0.05).

**Conclusions:**

The authors conclude that a micro-dose of resistance-exercise can reduce serological DHEA-S concentration within 60-min of exercise cessation. Additionally, mood-state assessment in untrained individuals can be considered for non-invasively indicating exercise-induced concentration changes in the stress biomarker, DHEA-S, providing prospects for the development of safer, more sophisticated exercise-monitoring practice.

## Background

The link between high-level athletic participation and adverse psychological and physiological events are well documented (Fitts 1994; Kellmann 2010). This has identified a need for improved athletic-monitoring procedures, and therefore, potential relationships between biology and psychology must be investigated and characterised. The neurosteroid dehydroepiandrosterone-sulfate (DHEA-S) and the pleitrophic cytokine interleukin-6 (IL-6) have recently been identified as potential regulators of fatigue and recovery from exercise (Liao et al. 2012; Robson-Ansley et al. 2004), providing an opportunity to compare biomarkers with affective parameters. A pitfall however with the existing data regarding the effect of exercise on these biomarkers relates to the dose of exercise which researchers have employed in their investigations to date. That is, only the influence of high-dose exercise on DHEA-S and IL-6 has been investigated, and additionally, existing studies have focused primarily on aerobic and mixed-type exercise modes (Lee et al. 2006; Liao et al. 2012; Robson-Ansley et al. 2004; Welc and Clanton 2013). Consequently, no data is available which describes the potential for low-dose exercise to alter DHEA-S and IL-6 profiles. Moreover, further data is also needed regarding the response profiles of DHEA-S and IL-6 to resistance-exercise specifically. In order to optimise exercise-monitoring practice, consequences of both low and high-dose exercise must first be understood.

An additional need for conducting low-dose exercise research into DHEA-S and IL-6 relates to maximising the standardisation of a researcher’s exercise protocol. Current scientific understanding of DHEA-S and IL-6 responses to exercise has been based on high-dose multiple-joint exercise protocols, where inter-individual differences in biomechanics [such as joint stability and sensorimotor control (Frank et al. 2013; Solomonow and Krogsgaard 2001)] may adversely affect a researcher’s ability to standardise their exercise protocols. If standardisation of the exercise performance was compromised in research, the validity of study findings could be adversely affected. Therefore, there is an evident need for low-dose exercise investigations which employ highly standardised exercise protocols, particularly when attempting to characterise biomarker and affective responses to exercise, as well as biological-affective relationships for the purpose of developing improved exercise monitoring practices.

Currently, a post-exercise reduction in DHEA-S has been a consistent finding in studies using high-volume exercise protocols [mixed-type and resistance based (Chen et al. 2011; Tsai et al. 2006)]. In weightlifters who demonstrated a post-exercise strength-decay and DHEA-S reduction, an increase in DHEA-S towards baseline during recovery was associated with improved strength-performance (Chen et al. 2011). The observed reduction in DHEA-S in response to exercise is believed to represent physiological consumption which promotes recovery (Gudemez et al. 2002; Lee et al. 2006). However, no study examining the DHEA-S response to resistance-exercise has measured DHEA-S levels within 3 h of exercise cessation. The induced elevation of baseline DHEA-S (through supplementation) has been reported to improve exercise-induced delayed onset of muscle-soreness (DOMS) (Liao et al. 2012). Additionally, DHEA-S levels have been linked to mood-state adjustment in elite golfers post major competition (Wang et al. 2009). Collectively these findings suggest that DHEA-S may have a broad role in recovery processes. Since elite-competition environments produce strong emotional alterations in athletes (Lane et al. 2007; Terry and Lane 2000) which could compromise the researcher’s control of variables, further research combining DHEA-S and mood indices are warranted in a controlled scientific environment.

The cytokine IL-6 has also been linked to performance and affective changes in athletes. The documented secretion of IL-6 from contracting skeletal muscle during and post high-volume exercise has been frequently reported (Welc and Clanton 2013), and of note, occurs in the absence of muscle damage (Castell et al. 1997; Gadient and Patterson 1999). Importantly, the release of IL-6 from skeletal muscle during and post-exercise appears to be augmented by exercise duration and intensity (Febbraio and Pedersen 2002; Ostrowski et al. 2000; Pedersen and Hoffman-Goetz 2000). Despite this consistent finding, no data is available which describes the response of IL-6 to low-dose exercise, thus a complete dose–response relationship (which could be useful in exercise monitoring) cannot yet be established. From a functional perspective, injection of IL-6 (at doses equivalent to those produced post-exercise) have been shown to increase perception of fatigue, reduce concentration, adversely affect global mood-state and significantly decrease 10 km time-trial performance in male runners (Robson-Ansley et al. 2004; Späth-Schwalbe et al. 1998). Multidisciplinary research is now needed which co-investigates the effect of resistance-exercise on DHEA-S, IL-6 and compares these markers with mood-state using a controlled exercise intervention such as thumb exertion. Since mood-state profiles can be easily administered in a non-invasive manner (compared with a blood-test), knowledge of potential relationships between mood-profiles and molecular markers associated with physiological fatigue (IL-6), stress and recovery (DHEA-S) will ultimately assist in developing safer, non-invasive monitoring techniques relevant to exercise-prescription.

To the best of the authors’ knowledge, the present study is the first to examine the effects of a micro-dose of resistance-exercise on DHEA-S and IL-6 profiles, in addition to the acute effect of resistance-exercise on serological DHEA-S. In the present study, the following hypotheses were made: (i) A micro-dose isometric thumb exertion will induce a serological decrease in DHEA-S concentration and a serological increase in IL-6 concentration observable within 60-min of exercise cessation; (ii) Post-exercise mood-state determinants will non-invasively indicate physiological stress changes evidenced through serological DHEA-S and IL-6 profiles.

## Methods

### Participants

Prior to recruitment of subjects, this study received full ethical approval from the Griffith University Human Research Ethics Committee. The study conformed to the ethical standards set out in the Declaration of Helsinki.

Before being admitted to the study, all participants were given a detailed explanation of the study procedures, experimental interventions and potential risks involved. Each participant then signed a written consent form.

Thirty apparently healthy men (18–40 years) voluntarily participated as subjects in the present study. These participants also acted as subjects in a previous study (Szlezak et al. 2015) conducted in our laboratory. Twenty men were weightlifting athletes who ranged from local to state-level competitors and trained at a frequency of two or more weightlifting sessions per week. The other ten men were untrained (not participating in any regular exercise and had not performed resistance-exercise for at least 12 months). The untrained subjects were grouped together into the untrained experimental group (UT_EXP_; n = 10). Using the concealed, third party randomisation method described previously (Schulz et al. 1995), weightlifting athletes (n = 20) were randomly allocated into one of two groups: (i) Weightlifting experimental (WL_EXP_; n = 10); (ii) Weightlifting placebo (WL_PLA_; n = 10). All subjects were blinded to their group allocation. Subject characteristics are displayed in Table 1.

**Table 1:** Subject characteristics

Group	WL_PLA_	UT_EXP_	WL_EXP_
n	10	10	10
Age (year)	26 (±7)	27 (±7)	27 (±5)
Height (cm)	181 (±7)	179 (±8)	183 (±8)
Body mass (kg)	84.5 (±10.9)	81.6 (±10.6)	87.6 (±9.3)
Resistance-training sessions (per week)	4 (±1)	0 (±0)	4 (±1)
Hand dominance	R: n = 8	R: n = 9	R: n = 7
	L: n = 2	L: n = 1	L: n = 3
Lateral pinch (kg)	8.6 (±1.5)	8.6 (±1.5)	9.4 (±1.5)

Values are presented as mean ± standard deviation

### Experimental approach

The present study employed a single-blinded, randomised, placebo-controlled study design. To control for the possible effects of prior-physical exertion on study results (Chen et al. 2011), participants abstained from all forms of exercise for 48 h prior to their appointment. Each participant attended the testing laboratory on a single occasion only (temperature monitored at 23 °C). Participants were subjected to a pre-screening performed by a Doctor of Physical Therapy to confirm that they were apparently healthy. Participants were excluded based on: i) Past or present history of upper limb/hand musculoskeletal pathology, cardiovascular, metabolic, or respiratory illness; ii) Contraindications to resistance-exercise (Pollock et al. 2000). Participants were then familiarised with the intervention and assessment procedures to be used. Briefly, participants were shown a copy of the BRUMS-24 and instructed on how to complete it. Participants were also familiarised with the B&L Engineering pinch gauge dynamometer (PG-60; B&L Engineering, CA, USA), the testing procedure, and performed five sub-maximal pinch efforts separated by 30 s rest before immediately completing a maximal-effort practice session identical to the testing session (see MVC Assessment). This familiarisation process also ensured any pre-fatiguing effect associated with this familiarisation procedure was consistent among subjects.

Following familiarisation, each participant sat in the standardised testing chair where an intravenous (IV) catheter (BD Insyte Autoguard 22 GA 1.00 IN; Becton-Dickinson, NJ, USA) was inserted into the median cubital vein of the participant’s dominant hand and was left in situ for serial blood sampling. This sampling method promoted precise timing of blood collection and avoided repeat needle-trauma which could affect results. At each collection point, 5 ml of blood was drawn and then discarded before collecting sample blood, and catheter lines were maintained through saline flushing. Note that as catheter lines could contain traces of coagulated blood or saline after blood collection, it was considered necessary to discard the first 5 ml of blood obtained at each blood collection time-point. This process ensured that only fresh whole blood was collected for analysis. Blood was collected directly into an 8.5 ml serum separator tube (SST) Vacutainer (Becton-Dickinson, NJ, USA).

Following catheter insertion, participants rested for 10-min in the testing chair prior to collection of baseline measures to negate any affect the catheterisation could have on subsequent measures. Baseline measures were then recorded in the following order: (i) lateral pinch (see MVC Assessment); (ii) blood collection (see DHEA-S and IL-6 Analysis); (iii) mood-state profiling.

Following baseline testing, participants undertook either an exercise intervention (WL_EXP_ and UT_EXP_ groups) or a placebo intervention (WL_PLA_ group). Once the interventions were completed, all participants immediately underwent the following testing conditions: Blood sampling with subsequent mood-state analysis performed immediately (0-min), 10-, 20- and 60-min post the interventions. IV catheters were removed after the final collection point. All participants remained seated throughout the experiment and were instructed against moving the tested limb to avoid active-recovery and ensure protocol standardisation.

Note that each SST tube (Becton-Dickinson, NJ, USA) was inverted five times immediately post blood collection and in turn stood for 30-min at 23 °C to maximise the clotting effect of the tube (as per the manufacturer’s recommendations). Serum was subsequently extracted and stored at −20 °C for DHEA-S and IL-6 analysis at a later date (see *DHEA*-*S and IL*-*6 Analysis*).

### Procedures

#### MVC Assessment

Maximum voluntary contraction (MVC) lateral-pinch assessment occurred using a B&L Engineering pinch gauge (PG-60; B&L Engineering, CA, USA) which has reported reliability and validity (Mathiowetz et al. 1984, 1985, 2000). All subjects undertook this assessment with their dominant hand only. To eliminate testing bias, one tester gave instructions and reset the dynamometer and a separate tester viewed and recorded each test result. Subject encouragement was standardised by using previously reported instructions (Mathiowetz et al. 1984). Subjects were tested using the protocol (Mathiowetz et al. 1985) and standardised testing (anatomical) positioning previously described (seated in a chair with arm rests and full back support, the shoulder adducted to neutral and neutrally rotated, elbow flexed at 90°, forearm in the neutral position, and wrist between 0° and 30° dorsiflexion and between 0° and 15° ulnar deviation [Fess and Moran 1981; Mathiowetz et al. 1985]). This also controlled for contribution from accessory musculature. During testing, subjects were instructed to give a maximal effort. Immediately after the first effort was completed, the dynamometer was reset and a second (and final) effort was performed. Note that no rest occurred between efforts. Values were recorded and later averaged.

#### Exercise intervention

A previously described micro-dose exercise protocol (Szlezak et al. 2015) formed the exercise intervention. This involved undertaking a lateral pinch-manoeuvre in a standardised testing position (Fess and Moran 1981; Mathiowetz et al. 1985) and utilised a B&L Engineering pinch gauge (PG-60; B&L Engineering, CA, USA). The purpose of the lateral-pinch manoeuvre was to minimise the potential effects of inter-individual biomechanical variation and accessory muscle exertion on study results (Punsola-Izard et al. 2012). 4-min total-work (isometric thumb exertion) was performed across four 60-s work-intervals, each separated by one 60-s rest interval. The total time for the exercise intervention was 7-min. The initial two work intervals were performed at an intensity of 50 % MVC and the final two work intervals at an intensity of 35 % MVC (where MVC was determined during the MVC Assessment).

Participants constantly monitored the pinch gauge during the work-intervals to ensure they maintained the correct load (kg). The standardised command “keep the needle at your weight” (Szlezak et al. 2015) was voiced by a researcher if the applied load began to vary from that specified.

#### Placebo intervention

WL_PLA_ participants undertook a previously described placebo intervention (Szlezak et al. 2015) comprising of four 60-s placebo-intervals separated by 60-s rest-intervals. Specifically, WL_PLA_ subjects held the pinch gauge without applying any downward pressure and thus no exercise was performed. This was considered a placebo since participants had no means of determining if this was a micro-dose exercise intervention or not. Importantly, this placebo intervention also served as a control for potential time-dependant (non-exercise related) variation in outcome measures. Body positioning in the placebo intervention was identical to the exercise intervention, and participants rested during the rest-interval as described in the exercise intervention. To maintain consistency with the exercise intervention, 7-min total-duration elapsed during the placebo intervention.

#### DHEA-S and IL-6 analysis

Serum was extracted in SST tubes (Becton-Dickinson, NJ, USA) by centrifugation (1000 RCF × 10-min at 23 °C) in a swinging bucket centrifuge. Serum aliquots were then immediately stored at −20 °C and later thawed and analysed as described below.

DHEA-S was quantified using an enzyme-linked immunosorbent assay (ELISA) kit from Alpha Diagnostic International (San Antonio, Tx, USA). DHEA-S was analysed in serum collected at 10-, 20- and 60-min post the interventions. This observation window was selected for DHEA-S since the acute response to exercise is unreported.

IL-6 was quantified using a high-sensitivity ELISA kit (sensitivity of 0.03 pg/ml) from eBioscience (Vienna, AUT). A high sensitivity kit was selected due to the small muscle group being exerted in this study. IL-6 was analysed in serum collected at 0-, 10- and 20-min post the interventions. Hyper-acute measurement time-points were chosen in consideration of previously reported post-exercise profiles (Welc and Clanton 2013).

All standards, controls and samples were analysed in duplicate (Powell et al. 2015). Absorbance values were obtained using an automated microplate reader (Modulus Microplate, Turner BioSystems; Sunnyvale, CA, USA) and absorbance values which represented a duplication of a common sample were averaged. Absorbance values were converted to analyte concentrations (DHEA-S & IL-6 in μg/ml & pg/ml respectively) using specialised ELISA software (Hitachi Solutions America, Ltd, CA, USA) with a Five Parameter Logistics curve (Baud 1993). Intra-assay and inter-assay coefficients of variation (CV) were 5.4 and 4.8 % for DHEA-S respectively, and 7.1 and 6.0 % for IL-6 respectively. Of note, these values fell within the precision ranges specified by the kits’ manufacturers.

#### Mood-state profiling: BRUMS-24

Mood-state parameters, vigour, tension, and fatigue were assessed using the 24-item Brunel Mood Scale; a psychometric assessment with reported reliability, internal consistency, validity (across multiple sports) and brevity of administration (Hashim et al. 2010; Lan et al. 2012; Terry and Lane 2000; Terry et al. 2003). Scoring occurred using official methods described previously (Terry and Lane 2010). Whilst multiple mood-parameters could have been assessed, vigour, tension and fatigue were chosen to provide an overview of the stress-recovery dynamic associated with exercise which was hypothesised to be reflected in the chosen biomarkers, DHEA-S and IL-6, (Kellmann 2010; Robson-Ansley et al. 2004; Späth-Schwalbe et al. 1998; Terry and Lane 2000).

### Statistical analysis

Values presented are as mean ± standard deviation. IBM SPSS Statistics 22 was used for all statistical testing. Fully-factorial ANOVA with repeated measures was used to determine any interaction among, or main effect of, the independent variables; group and time. Where the assumption of Sphericity was violated, Greenhouse-Geisser adjustment was applied. Least squares difference pairwise comparisons were used to detect the specific site of any significant effect identified. Effect sizes were determined by calculating partial eta squared (η^2^) values. Pearson’s correlation coefficients were calculated to: (i) assess if age and baseline DHEA-S concentration were correlated; (ii) determine the relationships among DHEA-S levels and mood. Statistical significance was accepted at p < 0.05.

## Results

No statistical differences existed for age (F = 0.08; p = 0.93), height (F = 0.54; p = 0.59) and body mass (F = 0.86; p = 0.44) variables.

### DHEA-S

**Fig. 1:**
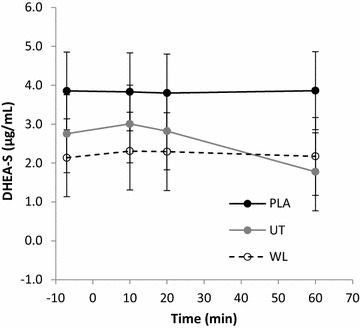
Serum DHEA-S concentration across time. PLA: WL_PLA_; UT: UT_EXP_; WL: WL_EXP_

Serum-DHEA-S concentrations did not differ between experimental groups at baseline, nor did those between WL_PLA_ and UT_EXP_ (p > 0.05). WL_EXP_ possessed a lower baseline concentration than WL_PLA_ (p = 0.03). A significant interaction between time and group was found for DHEA-S (F = 2.908, p = 0.033; η^2^ = 0.177). Pairwise Comparisons revealed no change in DHEA-S from baseline for either WL_PLA_ or WL_EXP_ at any post intervention time-point (p > 0.05). UT_EXP_ DHEA-S remained unchanged up to 20-min post-intervention (p > 0.05) then decreased (Δ36.9 %) from 20- to 60-min post-intervention (p < 0.01). At 60-min post-intervention, DHEA-S concentration was significantly below baseline (Δ35.3 %, p = 0.012). See Fig. 1.

### IL-6

No interaction was found between time and group (F = 1.546, p = 0.228; 32 % [observed power]) regarding IL-6 concentration in response to the group interventions. No differences existed among groups for IL-6 concentration at baseline (p > 0.05). No main effect of time was observed (F = 2.021, p = 0.164).

### Vigour

No interaction was found between time and group (F = 1.320, p = 0.267; 40 %) for vigour scores. WL_PLA_ and WL_EXP_ vigour scores did not differ at baseline (p > 0.05), nonetheless UT_EXP_ vigour was lower than other groups (p < 0.05). No main effect of time was observed (F = 2.456, p = 0.082).

### Tension

**Fig. 2:**
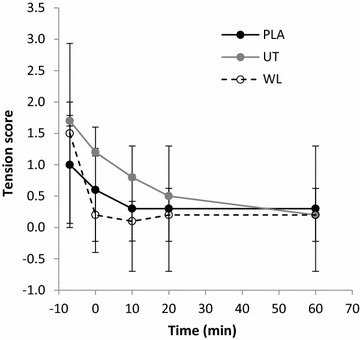
Tension score across time. PLA: WL_PLA_; UT: UT_EXP_; WL: WL_EXP_

No differences existed among groups for tension scores at baseline (p > 0.05). No interaction of time and group was found for tension (F = 0.944, p = 0.445; 20 %), however there was a main effect of time (F = 9.873, p = 0.000; η^2^ = 0.268). Pairwise Comparisons revealed that WL_PLA_ tension did not change from baseline across time (p > 0.05). UT_EXP_ tension remained unchanged at 0- and 10-min post-intervention (p > 0.05), before decreasing (Δ41.2 %) below baseline values by 20-min post-intervention (p = 0.017). Tension continued to decrease from 20- to 60-min post-intervention and was significantly below baseline at 60-min post-intervention (Δ100 %, p < 0.01). WL_EXP_ tension decreased below baseline immediately post-intervention (Δ86.7 %, p = 0.022) and remained decreased across time (p = 0.01). See Fig. 2.

### Fatigue

**Fig. 3:**
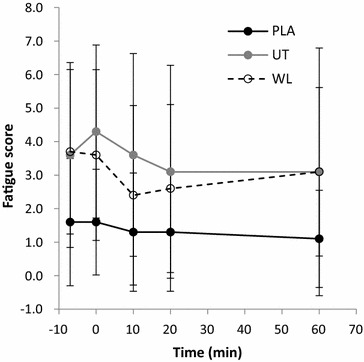
Fatigue score across time. PLA: WL_PLA_; UT: UT_EXP_; WL: WL_EXP_

No differences existed among groups for fatigue scores at baseline (p > 0.05). No interaction of time and group was found for fatigue (F = 0.765, p = 0.569; 25 %) however there was a main effect of time (F = 3.104, p = 0.045; η^2^ = 0.10). Pairwise Comparisons revealed that WL_PLA_ fatigue did not change from baseline across time (p > 0.05). UT_EXP_ fatigue had elevated from baseline (Δ11 %) immediately post-intervention yet this failed to reach significance (p > 0.05). By 20-min post-intervention, fatigue had decreased from the immediately-post value (Δ25 %) and this was highly significant (p < 0.01). At the final time-point (60-min post-intervention) fatigue remained lower than the immediately-post value (Δ22.5 %, p = 0.035) and was not different from baseline (p > 0.05). WL_EXP_ fatigue demonstrated a non-significant change immediately post-intervention (Δ3 %; p > 0.05). By 10-min post-intervention, fatigue had decreased significantly from the immediately-post value (Δ22.2 %, p < 0.01) and the value remained reduced at 20-min post-intervention (p = 0.015). Fatigue at the final time-point was not different from either immediately-post or baseline values (p > 0.05). See Fig. 3.

### Correlations

No correlation was observed between age and baseline DHEA-S (r = −0.26, p = 0.16). No correlations were found between changes in DHEA-S and: (i) *changes in tension* (WL_PLA_: r = 0.525, p = 0.119; UT_EXP_: r = −0.382, p = 0.276; WL_EXP_: r = −0.332, p = 0.349); (ii) *changes in fatigue* (WL_PLA_: r = −0.473, p = 0.167; UT_EXP_: r = 0.470, p = 0.170; WL_EXP_: r = 0.3, p = 0.4).

## Discussion

The primary finding of the present study was that a serological decrease in DHEA-S concentration occurs within 60-min succeeding a micro-dose of resisted thumb-exertion in untrained participants. This decrease is believed to represent physiological consumption for stress compensation (Lee et al. 2006; Gudemez et al. 2002) and this notion was supported by the unique changes observed in mood-state determinants, tension and fatigue, relative to the physical aptitude of participants in the present study. Additionally, the magnitude of change in DHEA-S matched previous reports for male subjects under much larger-dose conditions (Chen et al. 2011; Liao et al. 2012). These are highly novel observations considering the minimal dose of exercise employed, and the acute time-course in which changes occurred (Lee et al. 2006; Liao et al. 2012; Tsai et al. 2006). Importantly, the present study utilised a highly-standardised exercise-intervention (Mathiowetz et al. 1985; Punsola-Izard et al. 2012) which controlled for potential inter-individual differences in biomechanics. The present findings therefore validate and expand upon existing data on DHEA-S responses to exercise which were obtained through high-dose, multiple-joint exercise protocols (Chen et al. 2011; Lee et al. 2006; Liao et al. 2012; Tsai et al. 2006).

Throughout the present study, no change was observed for the WL_PLA_ group in any measure. Also, no change occurred in DHEA-S for the WL_EXP_ group. It is plausible that the short duration, micro-dose of exercise was insufficiently stressful to alter the physiological consumption rate of DHEA-S in trained subjects. Prior to the present study, the earliest measurement time-point of DHEA-S concentration following resistance-exercise was documented at 3 h (Chen et al. 2011), however no changes were observed until 24 h after exercise cessation. As the present study did not take measures beyond 60-min post-exercise, the DHEA-S response profile within 1–3 h post-exercise is unclear. It may be that low-dose physical exertion induces a more rapid reduction in DHEA-S than high-dose exertion which could have implications for recovery and exercise-prescription. What is evident however from the present study’s findings is the ability for a micro-dose of resisted physical exertion to disrupt hormonal homeostasis. Considering that a post-exercise reduction in DHEA-S likely represents physiological consumption which facilitates recovery (Lee et al. 2006; Gudemez et al. 2002), research is now needed which compares DHEA-S temporal responses to both low and high-dose exercise protocols. This research should incorporate mood-state measures like the present study to aid in interpreting the clinical implications of DHEA-S changes. This will ultimately assist in developing non-invasive exercise-monitoring techniques and assessments.

The present study was also the first to examine serological IL-6 response to a micro-dose of exercise. IL-6 release from skeletal muscle during and post-exercise has been consistently reported in the literature (Castell et al. 1997; Gadient and Patterson 1999; Welc and Clanton 2013) and its release appears positively influenced by exercise duration and intensity (Febbraio and Pedersen 2002; Ostrowski et al. 2000; Pedersen and Hoffman-Goetz 2000). Whilst no changes in IL-6 were observed in the present study, this finding empirically supports the notion of an exercise dose–response for IL-6 release. Considering that a high-sensitivity quantification was performed in the current study, the null IL-6 result is likely explained by the hypo-intensity and hypo-duration of the exercise intervention employed. It may be possible that skeletal muscle has an IL-6 ‘release-threshold’ which must be met before IL-6 is released to signal the onset of stress (Welc and Clanton 2013). This notion should now be investigated through experimental creation of a dose–response curve.

In addition to DHEA-S and IL-6, the present study investigated specific mood-state determinants associated with recovery. The negative result seen for vigour may be explained by the micro-dose of the employed exercise intervention. Whilst both experimental groups decreased to a similar magnitude in tension, WL_EXP_ decreased more rapidly than UT_EXP_. The UT_EXP_ delay in tension reduction suggests that the exercise-intervention placed greater psychological strain on the untrained participants than the trained. This notion is supported by the observed post-exercise reduction in serological DHEA-S (Gudemez et al. 2002; Lee et al. 2006). Whilst no correlations were found between *change in DHEA*-*S* and *change in tension*, combined DHEA-S and tension responses were unique among trained and untrained groups indicating a link between DHEA-S and mood as suggested previously in golfers (Wang et al. 2009). Further to tension, unique fatigue profiles were also observed for trained and untrained subjects, however no correlations were found between DHEA-S and fatigue profiles. With reference to fatigue levels recorded immediately post-exercise, subsequent fatigue values reduced more rapidly in WL_EXP_ than UT_EXP_. Like tension scores, this finding further suggests that the present study’s exercise-intervention induced a greater stress–response in the untrained versus trained subjects. More significantly, both fatigue and tension scores explain the increased physiological consumption of DHEA-S seen in the untrained subjects only. This relationship between mood parameters and DHEA-S serves as further evidence of the role of DHEA-S in recovery from resistance-exercise. Additionally, this demonstrates the potential for developing sophisticated non-invasive exercise monitoring tools once the links between biomarkers and determinants of mood have been further characterised.

## Conclusions

The present study is the first to demonstrate that a micro-dose of resisted thumb exertion induces a post-exercise decrease in serological DHEA-S. This reduction is believed to represent increased physiological consumption for stress compensation and recovery purposes (Gudemez et al. 2002; Lee et al. 2006) and occurred in untrained participants only. Of note, this finding highlights the importance for researchers to investigate low-dose exercise protocols in their research, since a micro-dose of resisted thumb exertion was able to distort hormonal homeostasis. Of additional novelty, the observed serological DHEA-S change occurred significantly earlier after the cessation of exercise than previously reported. This suggests that low-dose exercise may affect DHEA-S homeostasis more rapidly than high-dose exercise and warrants further investigation. No changes were observed in serological IL-6 suggesting that the present study’s micro-dose exercise-intervention was below the necessary dose/threshold to elicit a response. Further research is now needed which investigates if skeletal muscle has a ‘release threshold’ for IL-6. Unique changes in mood-determinants tension and fatigue, relative to the physical aptitude of participants supported the observed DHEA-S reduction in the untrained subjects only. This finding supports the role of DHEA-S in stress adjustment to resistance-exercise. Importantly, this relationship is preliminary evidence that mood-state assessment can be considered for non-invasively indicating biological stress–responses in an exercise setting, providing prospects for the development of safer, more sophisticated exercise monitoring practice.

## Abbreviations

ANOVA: analysis of variance
BRUMS-24: 24-item Brunel Mood Scale
DHEA-S: dehydroepiandrosterone-sulfate
ELISA: enzyme-linked immunosorbent assay
IL-6: interleukin-6
MVC: maximum voluntary contraction
SST: serum separator tube
